# Polyethylene glycol as a cause of anaphylaxis

**DOI:** 10.1186/s13223-016-0172-7

**Published:** 2016-12-13

**Authors:** Katharina Wylon, Sabine Dölle, Margitta Worm

**Affiliations:** Klinik für Dermatologie, Venerologie und Allergologie, Charité Universitätsmedizin Berlin, Charitéplatz 1, 10117 Berlin, Germany

**Keywords:** Anaphylaxis, Drug additives, Hypersensitivity, Macrogol, Polyethylene glycol

## Abstract

**Background:**

Polyethylene glycols (PEGs) or macrogols are polyether compounds and are widely used as additives in pharmaceuticals, cosmetics, and food.

**Case report:**

We report on a Caucasian patient experiencing recurrent severe allergic reactions to several drugs. An extensive diagnostic workup including skin prick tests, intradermal tests (IDT) and a double-blind oral challenge was performed to identify the trigger of anaphylaxis. In the present case hypersensitivity to the additive polyethylene glycol was confirmed by an IDT suggesting an Immunoglobulin E-dependent mechanism as a cause of the reaction.

**Conclusion:**

Potential life-threatening hypersensitivity reactions to hidden molecules like macrogol may be underdiagnosed. Cases of immediate-type PEG hypersensitivity were reported with increasing frequency. The awareness regarding the allergenic potential of PEG should be raised and a proper product labelling is crucial to prevent PEG mediated hypersensitivity.

## Background

Polyethylene glycol (PEG) or macrogol is a polyether compound. It is widely used as an additive in pharmaceuticals, cosmetics and food [1]. Different types of macrogol exist according to their molecular weight from 300 g/mol to 10,000,000 g/mol [2]. Anaphylactic reactions to macrogol are rarely reported. However, in recent years more reports appeared in the literature with macrogol induced hypersensitivities due to drugs, personal hygiene products, dental products, lozenges and lubricants [3, 4]. Here we report on a female with a history of three immediate type reactions triggered by macrogol 3350.

## Case report

A 46-year-old Caucasian female with no known allergies received an intraarticular injection with a local anesthetic (Xylonest^®^: prilocaine, sodium chloride, sodium hydroxide/hydrochloric acid 7%). Eight hour after the injection she experienced nausea and a generalized pruritus. The symptoms resolved the next day without any medical treatment. One day later the patient was given an injection with medroxyprogesteronacetate (Clinovir^®^: medroxyprogesteronacetate, methyl-4-hydroxybenzoate and propyl-4-hydroxybenzoate, macrogol 3350, polysorbate 80, sodium chloride) from her gynecologist. Five minutes after the injection she developed generalized pruritus, sneezing, nausea and tachycardia. Previous injections with medroxyprogesteronacetate and prilocaine were well tolerated.

One year later the patient received an injection to treat a lumbar intervertebral disc prolapse containing lidocaine, bupivacaine hydrochloride, triamcinolone acetonide (Triamhexal^®^: triamcinolonacetonide, benzyl alcohol, macrogol 4000, sodium chloride, sodium dihydrogen phosphate-dihydrate, sodium hydrogen carbonate, polysorbate 80). Within 5 min the patient developed systemic anaphylactic symptoms, including pruritus, nausea, tachycardia and flush.

The patient presented to our outpatient clinic 4 months after the first incident and 3 days after the third reaction. We initiated an allergological work-up including skin prick tests with macrogol, Clinovir^®^, latex, benzyl alcohol, paraben mix, sodium-benzoate, p-hydroxybenzate acid, sodium-metabisulfite, local anesthetics (procaine 1%, lidocaine 1%, bupivacaine 0.5%, prilocaine 1%, articaine 1%, mepivacaine 1%, scandicaine 1%, xylocaine 1%, ultracaine 1%, novocaine 1%), glucocorticosteroids (dexamethasone, prednisolone, triamcinolone, methylprednisolone) which were negative for all tested substances (Table 1). A negative control (sodium chloride 0.9%) and a positive control (histamine 10 mg/ml) were included. Total immunoglobulin E (IgE) was 93.5 kU/l and tryptase was 1.8 µg/l. A double-blind, placebo-controlled oral challenge with the additives which are in the preparations of Clinovir^®^ and Triamhexal^®^ (sodium-benzoate 250 mg and p-hydroxybenzoic acid 250 mg) was negative (Table 2). An intradermal test was performed with local anesthetics and additives of the suspected trigger substances (1% macrogol 3350, 10% macrogol 3350, polysorbate 80, scandicaine, xylocaine, ultracaine, bupivacaine, prilocaine, novocaine). After injecting 1% macrogol 3350 intradermal a 6 mm wheel was seen. 15 min later we carried out a second intradermal test with 10% macrogol 3350 which showed a 6 mm wheel diameter (Table 3). Consecutively the patient developed a systemic reaction with generalized pruritus and a generalized urticaria. The wheel diameter enlarged up to 12 mm, 5 min after the application.

**Table 1:** Skin prick test (interpretation after 15 min)

Negative control	Sodium chloride 0.9%	0 mm
Positive control	Histamine	4 mm
Local anesthetics	Procaine 1%, lidocaine 1%, bupivacaine 0,5%, prilocaine 1%, articaine 1%, mepivacaine 1%, scandicaine 1%, xylocaine 1%, ultracaine 1%, novocaine 1%	0 mm
Glucocorticosteroids	Dexamethasone, prednisolone, triamcinolone, methylprednisolone	0 mm
Others	Macrogol 3350 1%, macrogol 3350 10%, Depot Clinovir^®^, latex, benzyl alcohol 1%, paraben mix 16%, sodium-benzoate 5%, p-hydroxybenzate acid, sodium-metabisulfite	0 mm

Positive test result: wheel diameter >3 mm

**Table 2:** Double-blind oral challenge

Additives	Na-benzoate 250 mg, p-hydroxybenzoic acid 250 mg	Negative

**Table 3:** Intradermal test (interpretation after 15 min)

Negative control	NaCl	0 mm
Positive control	Histamine	4 mm
Additives	Macrogol 3350 1%	6 mm
	Macrogol 3350 10%	6 mm
	Polysorbate 80 1%	0 mm
Local anesthetics	Scandicaine 1%, xylocaine 1%, ultracaine 1%, bupivacaine 0,5%, prilocaine 1%, novocaine 1%	0 mm

Positive test result: wheel diameter >3 mm

Based on the history and the obtained skin test results our patient was diagnosed with an immediate type reaction to macrogol. We suspect that the first severe allergic reaction our patient experienced was not solely induced by prilocaine but another local anesthetic or an unknown additive containing macrogol. We prescribed an emergency kit containing an epinephrine auto-injector, a glucocorticosteroid and an antihistamine. The patient received an allergy pass and was instructed to avoid PEG analogues when taking new, over-the-counter drugs prescription drugs, personal hygiene products, dental products and other potentially PEG containing products.

Two years after the diagnosis of the hypersensitivity to macrogol the patient ingested WICK Medinait^®^ (paracetamol, dextromethorphan hydrobromide, doxylaminsuccinate, sucrose, glycerol, macrogol 6000, sodium citrate, sodium benzoate, potassium sorbate) treating a common cold. Again, she developed dyspnea and a generalized rash.

## Conclusions

However, data from the European anaphylaxis-registry with currently 7935 registered anaphylactic cases only three were induced by macrogol. These findings may imply that polyethylene glycol hypersensitivity is potentially life-threatening but probably underdiagnosed as many drugs and food items contain macrogol [4–6]. Handling patients with macrogol hypersensitivity can be challenging because of the extensive allergologic work up, the necessity of the physician’s expertise and the limited avoidance options because many drugs, including those used for the treatment of allergic reactions such as antihistamines may contain macrogol as an additive [5]. Therefore, specific product labeling and awareness is required. Patients should be educated about drugs which may contain PEGs, but also other products like lubricants or ultrasound gels. Our case indicated the need for an increased patient and physician awareness to the allergic potential of macrogol.

Concerning the mechanism of anaphylaxis mediated by PEGs different mechanisms have been proposed. Our case supports the assumption of cross-reactivity between PEGs of different molecular weights and polyethylene glycol analogues [7, 8]. Like other authors have previously shown, the positive intradermal test suggests an IgE-dependent mechanism, although no control tests were performed on healthy individuals to rule out unspecific reactivity [9]. However, even after a 1:10 dilution a positive intradermal test was observed. Other methods besides an oral challenge test to confirm the diagnosis may be by basophil activation test or western blot to show specific IgE binding [9].

In conclusion, cases of immediate-type PEG hypersensitivity are reported with increasing frequency, therefore, awareness of PEG’s allergenic potential should be raised and better product labeling should be discussed.

## Abbreviations

IgE: immunoglobulin E
IDT: intradermal test
PEG: polyethylene glycol
